# Occupational calling promotes career construction among medical students: empirical evidence for public health workforce reserve

**DOI:** 10.3389/fpubh.2026.1879126

**Published:** 2026-06-26

**Authors:** Liwen Liu, Lifeng Han

**Affiliations:** 1 School of Business, Macau University of Science and Technology, Macao, China; 2 School of Public Administration, Shandong Normal University, Jinan, China

**Keywords:** career construction, meaning in life, medical students, occupational calling, public health workforce reserve, teacher–student interaction

## Abstract

The sustainable development of a modern healthcare system relies not only on the provision of basic healthcare services but also on a stable reserve of future healthcare professionals. Medical students constitute an important source of the future healthcare workforce, and their career construction may influence the long-term stability of healthcare service provision. However, from the perspective of public health workforce development, existing empirical studies have primarily focused on external incentives, while relatively limited attention has been paid to the psychological factors shaping medical students’ career construction. This study examined the relationship between occupational calling and career construction among medical students and further tested the mediating roles of teacher–student interaction and meaning in life. A cross-sectional questionnaire survey was conducted among medical students from an undergraduate university and a vocational college in Zhuhai, China. After excluding invalid responses with missing or abnormal age information, 532 valid questionnaires were included in the final analysis. Validated scales were used to measure occupational calling, teacher–student interaction, meaning in life, career construction, and self-efficacy. The results showed that occupational calling significantly predicted teacher–student interaction, meaning in life, and career construction. Teacher-student interaction has a positive predictive effect on meaning in life and career construction. At the same time, the mediating analysis indicates that teacher-student interaction and meaning in life exert significant mediating effects, jointly influencing the relationship between occupational calling and career construction. These findings suggest that strengthening occupational calling, improving teacher-student interactions, and enhancing meaning in life are crucial for cultivating public health professionals and promoting career construction among medical students. Therefore, medical education should not only focus on increasing the number of future healthcare professionals but also strive to support their career development and foster their long-term commitment to serving the healthcare system.

## 1 Introduction

Healthcare workers are central to the functioning of any health system (1). The WHO has emphasized that health systems depend on the availability, accessibility, acceptability, and quality of health personnel (2). In China, public hospitals undertake a major share of healthcare service delivery, and physicians in these institutions remain the main providers of outpatient and clinical services (3, 4). However, prolonged workload, occupational burnout, and weakened professional commitment may threaten the stability of healthcare service provision and the long-term sustainability of the health workforce (5). For this reason, the training of future healthcare professionals should not be viewed only as an issue within medical education, but also as part of broader public health workforce development (6).

Medical students are an important source of future healthcare professionals. Their current career construction, clarity of career goals, and willingness to remain in the medical profession may influence the future supply and stability of healthcare services (7). Previous studies on turnover intention and occupational stability in the healthcare workforce have mainly focused on practising healthcare workers, with attention to factors such as job security (8), healthcare policy environments (9), and job satisfaction (10). While these studies are valuable, they pay limited attention to the developmental stages individuals go through before formally entering the profession (11). Medical students are not only learners in medical education but also a potential pool of talent for the healthcare workforce; exploring the factors that influence their career development may provide valuable insights for strengthening medical education and supporting the long-term development of the public health workforce.

Occupational calling is a key concept in occupational psychology. It typically refers to an individual’s perception that their current or future profession is meaningful, has clear goals, and is closely aligned with their personal values (12). Some studies further emphasize that occupational calling reflects passion for one’s profession and a strong sense of meaning in one’s work (13, 14). For medical students, this concept is particularly important because the medical career is closely linked to life, health, social service, and ethical responsibilities. Therefore, a stronger sense of occupational calling helps students understand the value of medical work and establish a clearer direction for their future career.

However, the relationship between occupational calling and career construction may not be achieved through a single pathway. In medical education, teacher-student interactions serve as a vital source of external support. Through formative feedback, career guidance, role modeling, and emotional support, faculty can help students understand the social value and professional responsibilities of medical practice, thereby supporting their career exploration, planning, and engagement (15). At the same time, meaning in life may function as an internal psychological resource. Students who are able to link personal growth with patient care, social contribution, and public health responsibilities are more likely to form clear career goals and maintain a stronger sense of professional commitment (16, 17). Therefore, teacher-student interactions and meaning in life may help explain how occupational calling facilitates career construction among medical students.

This study makes three major contributions. First, it examines medical students’ career construction from the perspective of building the public health workforce. Rather than viewing career construction solely as an individual issue, this study links it to the training of future healthcare professionals. In this sense, medical students’ occupational calling and career construction are not only relevant to their personal growth but also to the long-term stability of the healthcare workforce. Second, this study constructs a chain mediation model that links occupational calling, teacher-student interactions, meaning in life, and career construction. This model organically integrates external educational support with internal meaning-making processes, indicating that occupational calling may not only directly influence career construction but also exert its effects through teacher-student interactions and meaning in life. Third, based on survey data from 532 medical students, this study provides empirical evidence of the association between occupational calling and career construction. The findings help elucidate the roles of teacher-student interactions and meaning in life in medical students’ career development and may offer valuable insights for medical education and the development of the public health workforce.

## 2 Theoretical foundation and research hypotheses

### 2.1 Theoretical foundation

This study is primarily grounded in career construction theory and social learning theory. Career construction theory, based on McAdams’s theory of the psychological self, explains from a life-span developmental perspective how individuals internalize their career self-concept into work roles, how they cope with career transitions, and how they manage their own careers (18). From this perspective, career construction is not only a process of planning future career development but also a process through which individuals align their personal values with future work roles. For medical students, a deep understanding of the meaning of the medical profession helps them form a relatively stable career development direction. Occupational calling can help students better understand the significance of their future careers, encourage them to explore future career directions more actively, and provide an intrinsic psychological foundation for career development.

Social learning theory explains the mediating pathway between teacher-student interaction and meaning in life. This theory posits that an individual’s learning stems not only from direct experience but can also be achieved by observing others’ behaviors and forming new cognitions (19). From this perspective, medical students acquire only foundational knowledge from textbooks, while most of their professional understanding must be developed through practice. Instructors not only convey knowledge but also play a crucial role as practical mentors. Therefore, high-quality teacher-student interactions can further help medical students understand their professional responsibilities and promote their career development. At the same time, meaning in life provides another important psychological pathway. When medical students are able to link their personal growth to patient care and social responsibility, they are more likely to develop a stronger sense of life meaning. Guided by this psychological state, they may establish clearer career goals and maintain a more consistent level of commitment throughout their career development.

Taken together, career construction theory and social learning theory suggest that a sense of professional mission may influence medical students’ career development through external educational support and internal meaning-making processes. Based on this logic, this study proposes a chained mediation model in which occupational calling promotes career construction through teacher-student interactions and meaning in life.

### 2.2 Research hypotheses

Occupational calling is closely linked to career construction. Individuals with a stronger sense of occupational calling are more likely to view their future work as meaningful and aligned with their personal values, which may enhance their motivation to explore and prepare for their careers (20). In the context of medical education, occupational calling helps students connect their future careers with social contribution, personal growth, and professional responsibility. Previous research also indicates that occupational calling can enhance career construction, and its positive effects are more readily evident in supportive environments that emphasize growth, adaptability, and mentorship (21, 22). Therefore, occupational calling may provide an important psychological foundation for medical students’ career construction. Based on this, the present study proposes the following hypotheses:

*H1*: Occupational calling is positively associated with career construction.

Interaction between teachers and students may be a key pathway through which occupational calling supports career construction. Students with a stronger occupational calling typically exhibit greater motivation to learn and are more willing to seek guidance from teachers. In medical education, teachers not only provide academic guidance but also offer feedback, emotional support, career counseling, and serve as professional role models. According to social learning theory, students can cultivate professional attitudes and behavioral orientations by observing and learning from faculty members’ behaviors (23). Through such interactions, medical students may develop a clearer understanding of professional responsibilities, ethical requirements, and future career paths, thereby strengthening their career construction (24, 25). Based on this, the present study proposes the following hypothesis:

*H2*: Occupational calling is positively associated with teacher–student interaction.

*H3*: Teacher–student interaction is positively associated with career construction.

*H4*: Teacher–student interaction mediates the relationship between occupational calling and career construction.

Meaning in life may also serve as a mediator between occupational calling and career construction. Medical education involves not only the acquisition of professional knowledge and clinical skills but also the development of values such as a sense of responsibility, altruism, and a spirit of service. Students with a stronger occupational calling are more likely to connect their personal interests with the social value of medical work, which may deepen their appreciation of the meaning in life (26). Previous research indicates that occupational calling helps medical students cope with academic stress, clinical challenges, and the emotional demands of medical training (17, 27). When students perceive medical work as meaningful, they are more likely to define clear career goals and maintain sustained commitment throughout their career construction. Therefore, meaning in life may serve as a crucial psychological mechanism linking occupational calling to career construction. Based on this, the present study proposes the following hypothesis:

*H5*: Occupational calling is positively associated with meaning in life.

*H6*: Teacher–student interaction is positively associated with meaning in life.

H7: Meaning in life mediates the relationship between occupational calling and career construction.

**Figure 1:**
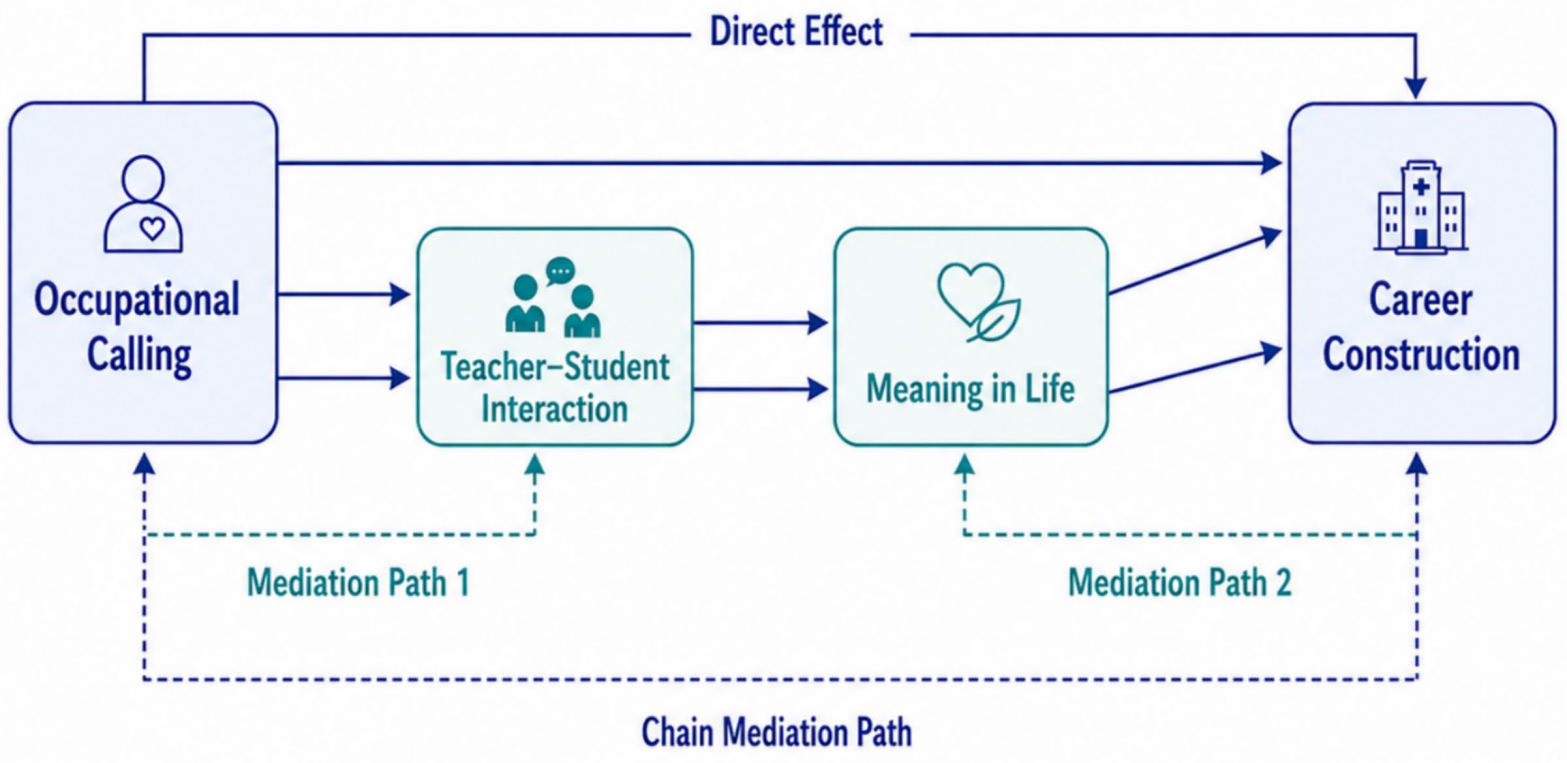
Research hypothesis model diagram.

Teacher–student interaction and meaning in life may interact with one another rather than functioning as two independent mechanisms. Through career guidance, emotional support, and the example set by professional role models, teachers can help medical students better understand the social value and ethical responsibilities of the medical profession, which in turn may enhance their meaning in life. In this process, occupational calling may initially motivate students to engage in teacher-student interaction, and these interactions may subsequently enhance their meaning in life, ultimately promoting their career construction. Based on this, this study proposes the following hypothesis (see Figure 1):

*H8*: Teacher–student interaction and meaning in life sequentially mediate the relationship between occupational calling and career construction.

## 3 Research methods

### 3.1 Study design and sample

This study employed a cross-sectional questionnaire survey design. The study participants were medical students from vocational schools and undergraduate institutions, including Zhuhai Health School and the Zhuhai Campus of Zunyi Medical University. The raw data comprised a total of 825 valid questionnaires. After data cleaning—which involved removing records with clearly invalid or unidentifiable age fields—a valid sample of 821 participants was obtained for formal.

This study was approved by the Academic Ethics Committee of the University of Science and Technology of Macau. All participants provided informed consent prior to completing the questionnaire. The questionnaire was collected anonymously, and participants were free to participate voluntarily and withdraw at any time. As a non-interventional survey, the study did not involve any additional risks.

### 3.2 Measuring tools

This study drew on the occupational calling scale developed by Dik et al. (28), the teacher–student interaction scale developed by Jafari and Asgari (29), the Student Career Construction Scale revised by Jiang et al. (30), and the Meaning in Life Questionnaire developed by Steger et al. (31). Since the existing scales were originally designed for the study of single variables, many items are used to verify specific situations. Therefore, the level of multicollinearity among the scale items cannot be strictly limited. However, this study also screened items with relatively high multicollinearity and finally formed a questionnaire scale including 12 items for occupational calling, 3 items for teacher-student interaction, 5 items for meaning in life, and 20 items for career construction.

## 4 Research findings

### 4.1 Data cleaning and sample characteristics

A total of 842 questionnaires were collected for this study. To ensure the validity and reliability of the data, the raw data from the questionnaires were cleaned. Exclusion criteria included: missing age information, non-numeric entries for age, response times under 40 s, questionnaires where all core items were answered with the same option, and questionnaires where more than 95% of items were answered with the same option. As shown in Table 1, after cleaning, there were 532 valid responses, resulting in a response rate of 63.18%.

**Table 1:** Data cleaning and valid sample size.

Item	Number	Percentage
Original questionnaires	842	100.00%
Excluded due to invalid age information	21	2.49%
Excluded low-quality responses	289	34.32%
Final valid sample size	532	63.18%

The demographic variables included in the survey are shown in Table 2.

**Table 2:** Demographic characteristics of participants.

Variable	Category	*n*	%
Institution	Zhuhai Health School	288	54.14
	Zunyi Medical University Zhuhai Campus	244	45.86
Gender	Female	425	79.89
	Male	107	20.11
Academic year	Vocational third year	170	31.95
	Third-year undergraduate	92	17.29
	Fourth-year undergraduate	88	16.54
	Second year	66	12.41
	Master’s student	64	12.03
	First year	52	9.77
Major	Nursing	186	34.96
	Pharmacy-related	111	20.86
	Clinical medicine	61	11.47
	Bioengineering	55	10.34
	Rehabilitation technology	43	8.08
	Other majors	76	14.29
Age	Mean ± SD	19.05 ± 2.92	
	Range	14–30	

For each of the core variables, the descriptive statistical analysis of occupational calling, teacher-student interaction, career construction, and meaning in life was conducted to obtain the preliminary understanding of their distribution characteristics. The average score for the core variables is given in Table 3.

**Table 3:** Descriptive statistics of main variables.

Variable	*N*	Min	Max	Mean	Std. dev.	Median
Occupational calling	532	1.00	5.00	3.494	0.766	3.500
Teacher–student interaction	532	1.00	5.00	3.840	0.872	4.000
Career construction	532	1.45	5.00	3.895	0.654	3.900
Meaning in life	532	1.00	5.00	3.823	0.738	4.000

### 4.2 Covariance analysis

In this study, SPSS 23.00 software was used to calculate the VIF for the data to quantify the degree of covariance and test for linear correlations among the independent variables. If the VIF value is too high, it indicates a serious problem of multicollinearity, which may lead to unstable parameter estimates in regression analysis and render the data unsuitable for further processing (29). As shown in Table 4, the VIF values for the “Occupational calling” “Meaning in life” and “Career construction” items were all below 5, indicating that multicollinearity at the item level was within an acceptable range. For the “Teacher–student interaction” items, one VIF value was below 5, while the other two were slightly above 5; however, the tolerance values were all greater than 0.1. Although some item-item correlation existed, it did not reach the level of severe multicollinearity, which may be related to the similarity among items in this short scale. Therefore, based on the final calculations, it can be concluded that this study does not have a multicollinearity issue. The questionnaire data are valid and reliable and can be analyzed in subsequent data analysis steps.

**Table 4:** Item-level multicollinearity analysis.

Construct	Item	VIF	Tolerance	Interpretation
Occupational calling	OC1	3.576	0.280	Acceptable
	OC2	4.791	0.209	Acceptable
	OC3	3.871	0.258	Acceptable
	OC4	3.914	0.255	Acceptable
	OC5	3.850	0.260	Acceptable
	OC6	4.568	0.219	Acceptable
	OC7	4.588	0.218	Acceptable
	OC8	3.776	0.265	Acceptable
	OC9	3.084	0.324	Acceptable
	OC10	3.586	0.279	Acceptable
	OC11	2.938	0.340	Acceptable
	OC12	2.600	0.385	Acceptable
Teacher–student interaction	TSI1	4.106	0.244	Acceptable
	TSI2	5.449	0.184	Slightly high
	TSI3	5.620	0.178	Slightly high
Meaning in life	MIL1	4.109	0.243	Acceptable
	MIL2	3.951	0.253	Acceptable
	MIL4	3.502	0.286	Acceptable
	MIL5	3.891	0.257	Acceptable
	MIL7	4.073	0.246	Acceptable
Career construction	CC1	2.945	0.340	Acceptable
	CC2	3.519	0.284	Acceptable
	CC3	3.851	0.260	Acceptable
	CC4	3.536	0.283	Acceptable
	CC5	3.432	0.291	Acceptable
	CC6	4.025	0.248	Acceptable
	CC7	2.767	0.361	Acceptable
	CC8	3.122	0.320	Acceptable
	CC9	3.886	0.257	Acceptable
	CC10	4.299	0.233	Acceptable
	CC11	2.953	0.339	Acceptable
	CC12	3.856	0.259	Acceptable
	CC13	4.373	0.229	Acceptable
	CC14	3.929	0.255	Acceptable
	CC15	4.808	0.208	Acceptable
	CC16	4.321	0.231	Acceptable
	CC17	3.841	0.260	Acceptable
	CC18	4.650	0.215	Acceptable
	CC19	4.581	0.218	Acceptable
	CC20	4.068	0.246	Acceptable

At the same time, to further validate the validity of the research questionnaire, this study reviewed the literature on multicollinearity. Some studies have suggested that, for questionnaires with a single variable, combining multicollinear variables into a single variable is an effective solution (32). By combining various items in the questionnaire, this study calculated the VIF values for each variable, as shown in Table 5. The results indicate that after merging multiple items into a single variable, all VIF values in the study model are less than 5, indicating that there is no multicollinearity.

**Table 5:** Construct-level multicollinearity analysis.

Construct	VIF	Tolerance
Occupational calling	1.597	0.626
Teacher–student interaction	1.518	0.659
Career construction	3.189	0.314
Meaning in life	2.935	0.341

### 4.3 Correlation analysis

Pearson correlation analyses were conducted to examine the preliminary relationships among occupational calling, teacher–student interaction, meaning in life, and career construction. The results are presented in Table 6.

**Table 6:** Correlations among main variables.

Variable	1	2	3	4
Occupational calling	1			
Teacher–student interaction	0.550^**^	1		
Career construction	0.474^**^	0.435^**^	1	
Meaning in life	0.425^**^	0.365^**^	0.811^**^	1

^**^p < 0.01.

As shown in Table 5, occupational calling, teacher–student interaction, meaning in life, and career construction were all significantly and positively correlated at the 0.01 level. Occupational calling was positively associated with career construction, teacher–student interaction, and meaning in life, suggesting that students with stronger occupational calling tend to report better career construction, more positive teacher–student interaction, and a stronger sense of meaning in life. Teacher–student interaction was also positively correlated with career constructionand meaning in life, indicating that supportive educational interaction may be related to students’ career preparation and meaning-related experiences. In addition, meaning in life showed a high positive correlation with career construction, suggesting a close association between students’ meaning-related psychological resources and their career construction.

### 4.4 Structural path analysis

Drawing from correlation analysis, this study further develops structural equation modeling to examine the path relations of occupational callin, teacher-student interaction, meaning in life and career construction. The coefficients related to the directional path are shown in in Table 7.

**Table 7:** Structural path coefficients.

Path	*β*	SE	*t*	*p*
Occupational calling → Teacher–student interaction	0.550	0.036	15.148	<0.001
Occupational calling → Meaning in life	0.321	0.046	6.921	<0.001
Teacher–student interaction → Meaning in life	0.188	0.046	4.059	<0.001
Occupational calling → Career construction	0.104	0.030	3.425	<0.001
Teacher–student interaction → Career construction	0.113	0.030	3.817	<0.001
Meaning in life → Career construction	0.725	0.027	26.527	<0.001

As shown in Table 7, occupational calling had a significant positive effect on teacher–student interaction, with a standardized path coefficient of *β* = 0.550. Thus, medical students with stronger occupational calling tended to show more positive teacher–student interaction and receive more career guidance and developmental support from teachers. The result suggests that occupational calling can encourage students to seek support and engage in career exploration.

Occupational calling also had a significant positive effect on meaning in life, indicating that it can enhance students’ understanding of the meaning of life and the value of the medical profession. Since medicine is closely related to social responsibility and care for life, students with stronger occupational calling are more likely to develop a stable sense of meaning in life.

Teacher–student interaction had a significant positive effect on meaning in life. This suggests that teachers’ career guidance, constructive feedback, and role modeling can help students further understand the meaning of medical study and their future careers. Teacher–student interaction is therefore not only a form of external educational support, but also a source of value guidance and meaning construction.

In the predictive pathways of career construction, occupational calling, teacher–student interaction, and meaning in life all had significant positive effects. Specifically, the direct effect of occupational calling on career construction was *β* = 0.104, the direct effect of teacher–student interaction was *β* = 0.113, and the effect of meaning in life on career construction was the largest, *β* = 0.725. The results indicate that occupational calling can still directly promote career construction after controlling for teacher–student interaction and meaning in life, although the effect is reduced. This implies that teacher–student interaction and meaning in life play important mediating roles in the relationship between occupational calling and career construction.

**Figure 2:**
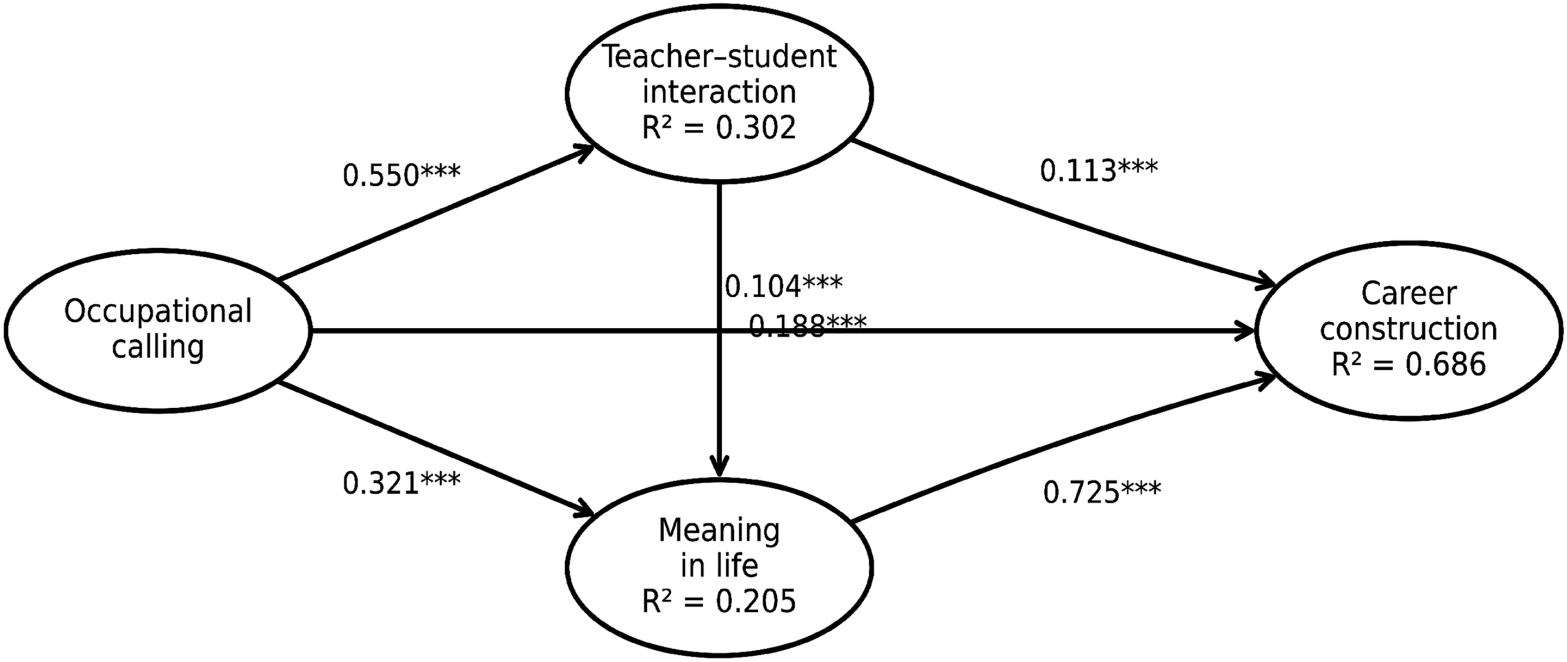
Model structure diagram.

Using the sense of career mission as the independent variable, career construct as the dependent variable, and teacher-student interaction behaviour and sense of meaning in life as the mediating variables. The results, as shown in Figure 2, showed that the correlation between the sense of career mission and career construct was significant, and the chain mediation effect between teacher-student interaction behaviours and the sense of meaning in life was significant.

### 4.5 Mediation effect analysis

The study utilized a bootstrap approach with 5,000 repetitions to examine the mediating role of teacher-student interaction and a sense of life’s meaning to further analyze the relationship between occupational calling and career construction. A 95% confidence interval that does not overlap with 0 indicates a significant mediation effect. The Table 8 contains the results.

**Table 8:** Bootstrap mediation effect analysis.

Mediating path	Effect	Boot SE	BootLLCI	BootULCI	*z*	*p*
Occupational calling → Teacher–student interaction → Career construction	0.062	0.024	0.018	0.114	2.554	0.011
Occupational calling → Meaning in life → Career construction	0.233	0.044	0.144	0.317	5.273	<0.001
Occupational calling → Teacher–student interaction → Meaning in life → Career construction	0.075	0.026	0.029	0.129	2.927	0.003
Total indirect effect	0.370	0.039	0.294	0.448	9.403	<0.001

As presented in Table 7, the Bootstrap confidence intervals for all three mediation paths do not contain 0, suggesting that all mediation effects are significant. The indirect effect through meaning in life was the largest, followed by the sequential pathway through teacher–student interaction and meaning in life. The total indirect effect was also significant, indicating that occupational calling influences career construction both directly and indirectly.

## 5 Policy implications for medical education and public health workforce development

This study offers important insights for medical education and the development of public health professionals. The findings indicate that occupational calling is positively correlated with career construction. This suggests that fostering occupational calling among medical students helps them better understand the significance and responsibilities of the medical profession, motivates them to set personal career goals, and links their individual career development to their roles within the healthcare system, thereby strengthening the potential pool of public health professionals.

At the same time, the mediating role of teacher-student interaction further indicates that students’ career construction is influenced not only by their intrinsic motivation but also by their educational environment. Therefore, medical schools should strengthen the mentorship system, move beyond the limitations of large-class lectures, establish small-group teaching and discussion sessions, and encourage faculty to participate more actively in students’ career construction. These measures will help students better understand the responsibilities of medical practice and clarify their career development paths.

Furthermore, meaning in life plays a significant role. During medical education, students should be encouraged to participate in clinical practice so they can experience the professional significance of their work firsthand. This helps students translate occupational calling into clearer career goals and maintain a more enduring commitment to their profession.

Overall, the training of future healthcare professionals should not be viewed solely in terms of enrollment size or the number of practitioners. Greater attention should be paid to the quality of medical students’ professional development during their education. By reinforcing a sense of professional mission, improving faculty-student interaction, and enhancing meaning in life, medical schools can better support students’ career development and contribute to the cultivation of a more stable and proactive public health workforce.

## 6 Conclusion

Based on survey data from 532 students at two medical schools, this study examined the relationships among occupational calling, teacher-student interaction, meaning in life, and career construction. The results indicate that occupational calling is positively correlated with career construction, while teacher-student interaction and career construction mediate this relationship.

Occupational calling provides an important psychological foundation for medical students’ career construction. Students who have a deeper understanding of the social value, ethical responsibilities, and public health significance of the medical profession are more likely to engage in career planning, exploration, and professional preparation. Faculty-student interactions further serve as an external educational mechanism. Effective interactions with faculty help students understand career development pathways, professional responsibilities, and the practical demands of medical practice. Life meaning represents an internal psychological mechanism. When medical students link their personal growth to patient care, social contribution, and public health responsibilities, they are more likely to form clear career goals and maintain sustained career commitment.

This study also reveals a chain mechanism: occupational calling promotes teacher-student interaction, which in turn reinforces meaning in life, and meaning in life subsequently supports career construction.

The following limitations should be noted. First, this study utilized cross-sectional survey data; therefore, it is not possible to draw strict inferences regarding causality. Future studies could employ a longitudinal design to examine changes in professional mission, teacher-student interactions, life meaning, and career construction over time. Second, the sample was drawn from two medical institutions in Zhuhai, which limits the generalizability of the findings. Future research should include medical students from different regions, institutions of varying levels, and diverse educational backgrounds. Finally, this study relies on self-administered questionnaire data. Future research could combine survey data with interview data, student records, or employment tracking data to better examine the long-term relationship between medical education and the development of the public health workforce.

## Data Availability

The raw data supporting the conclusions of this article will be made available by the authors, without undue reservation.
